# Kinetics of Immunoglobulins in Septic Shock Patients Treated With an IgM- and IgA-Enriched Intravenous Preparation: An Observational Study

**DOI:** 10.3389/fmed.2021.605113

**Published:** 2021-03-01

**Authors:** Giorgio Berlot, Alice Scamperle, Tatiana Istrati, Roberto Dattola, Irene Longo, Antonino Chillemi, Silvia Baronio, Giada Quarantotto, Silvia Zanchi, Erik Roman-Pognuz, Mattia Bixio, Ariella Tomasini

**Affiliations:** ^1^Department of Anesthesia and Intensive Care, Cattinara Hospital, University of Trieste, Trieste, Italy; ^2^Department of Anesthesia and Intensive Care, San Martino Hospital, Genova, Italy

**Keywords:** septic shock, immunoglobulins (G&M) IgG, IgM, infections, plasma concentration

## Abstract

**Objective:** To assess the variations of the blood levels of immunoglobulins (Ig) in septic shock patients treated with an Ig preparation enriched in IgM and IgA (eIg).

**Design:** The blood levels of Ig in survivors (S) and non-survivors (NS) of a group of septic shock patients were measured before the initial administration (D0) and 1 (D1), 4 (D4), and 7 (D7) days thereafter. The SAPS II score, the capillary permeability, the primary site of infection, the antibiotic appropriateness, and the outcome at 28 days were also assessed.

**Results:** In the interval D0–D7, the IgM increased significantly only in the S while remained stable in NS; the IgA significantly increased in both groups; the IgG did not vary significantly in both groups. At D4, the capillary permeability significantly decreased in S but not in NS.

**Conclusions:** The kinetics of the different classes of Ig after eIg were different between S and NS. This could be related either to (a) different capillary permeability in the two groups or to (b) higher Ig consumption in NS. Further studies to confirm the benefits of eIg in the treatment of sepsis syndrome and to define the specific target population and the correct eIg dose are warranted.

## Introduction

Despite different studies and meta-analyses demonstrating a better outcome in septic patients who received intravenous immunoglobulins (IvIg) (1–3) compared with controls, the Surviving Sepsis Campaign's guidelines (SSC) recommend against their use due to the lack of trials fulfilling the Evidence-Base Medicine criteria (4). The main criticisms are based on (a) patient-related variables, including the number and severity of comorbidities, the underlying immunitary capabilities, and the adequacy of other concomitant therapies; (b) the presence and the amount of antibodies against the responsible germ(s), including Multiple Drug-Resistant (MDR) strains in the preparation administered; and, finally, (c) the class of IvIg used (4, 5). Currently, just one preparation is enriched with elevated concentrations of IgM and IgA (eIg) (12% each), whereas the other ones contain these Ig in trace amounts only.

As far as this latter issue is concerned, it appears that the positive effects of IvIg are more marked in patients treated with eIg than with IgG only (6–8). Different factors could account for this finding, including the IgA-associated protective effects on the gut mucosa (9–11) and the pentameric structure of the IgM that allows their binding to different antigens located on the germ surface or to pathogen-associated molecular patterns (PAMP) with the subsequent activation of the complement (12, 13); moreover, IgM molecules exert an immumodulatory effect by scavenging excessive complement factors and blunting the production of some sepsis mediators (14, 15).

Besides the above listed confounding factors, another critical point associated with the IvIg is represented by the target blood values to achieve (16, 17). This could be particularly relevant in the case of eIg due to the high cost of the only preparation available. Actually, the manufacturer's indications recommend a standard dose of 250 mg/kg of body weight for 3 consecutive days and the choice to add further doses or not is left to the physician, independently from the initial and end-treatment blood values of the γ-globulins. This approach carries the inherent risk of over- or under-administration of the eIg and is far from the concept of precision medicine.

As some investigations demonstrated that in different critical conditions the kinetics of endogenous Ig was associated with the outcome (16, 17), we measured the time course of these substances in survivors and non-survivors of a group of septic shock patients before, during, and after the administration of eIg.

## Patients and Methods

This is a multicenter, observational, non-interventional study, involving two general adult Intensive Care Units (ICUs) (the Department of Anesthesia and Intensive Care of Trieste and the Department of Anesthesia and Intensive Care of Alessandria). As eIg (Pentaglobin®, Biotest, Dreiech, Germany) are routinely used in these ICUs and the study did not imply any randomization or interventions other than the standard medical care, the local Ethical committees were informed but considered the patients' informed consent unneeded (Trieste Ethical Committee statement 59/2015); moreover, according to the current policies, all medical records are freely available for review and/or research purposes provided that the data remain anonymous.

We included 54 patients admitted with septic shock in both ICUs. Septic shock was defined according to SEPSIS 3 criteria (18). Multiple drug-resistant germs (MDR) included methicillin-resistant *Staphylococcus aureus* and *Epidermidis*, extended spectrum beta-lactamase (ESBL) producing *Enterococcus faecium*, vancomycin-resistant Enterococci, MDR *Acinetobacter*, ESBL-producing and carbapemen-resistant Enterobacteriacae, and MDR *Pseudomonas*.

The overall treatment followed the SSC guidelines. Exclusion criteria were age <18 years, previous administration of IvIg, hematological tumors, known immune depression (i.e., AIDS), recent or ongoing treatment with immunosuppressant, preexisting chronic renal failure, and a life expectancy <3 months. The eIg were given at the dose of 250 mg/kg/day; the infusion lasted 10 h and was repeated for 3 days for a total dose of 750 mg/kg.

For all patients, we collected demographic characteristics, diagnosis at admission, and type of admission (surgical or medical). We used the SAPS score to assess the severity of the condition of each patient. We obtained blood samples for γ-globulins measurement immediately before starting the eIg infusion (D0) and successively after 1 (D1), 4 (D4), and 7 (D7) days. The different subclasses of IgG were not measured. The capillary leak index (CLI), calculated according to the formula: C-reactive protein (CRP) (milligrams/100 ml)/albumin (grams/liter) ^*^ 100 was considered a marker of capillary permeability at D0 and D4.

The primary site of infection, the antibiotics administered and their appropriateness, the microorganisms isolated, and the outcome at 28 days were also recorded. The adequacy of the antibiotics was assessed by comparing the drug administered with the results of the antibiograms, both at the ICU admission and later on.

The statistical analyses were performed using GraphPad Prism Version 8.0.2 (GraphPad Software, La Jolla, CA, www.graphpad.com) and R statistical package, software version 3.3.3. Descriptive statistics were analyzed for all the variables. Discrete variables were expressed as percentage and continuous variables were expressed as mean ± SD or median (25th−75th percentiles). Categorical variables were compared by chi-square or Fisher exact test. Continuous variables were compared using Student *t*-test. The variation of the blood levels of Ig was expressed as percentage, and non-parametric tests, such as Mann–Whitney, were used since the data were not normally distributed. A level of *P*-value <0.05 was considered statistically significant.

## Results

Overall, 54 patients were enrolled (Table 1); non-survivors at 28 days (NS = 14) had a significantly higher SAPS 2 score as compared with survivors (S = 40). According to our policy, the eIg were administered within 12 h from the diagnosis of septic shock (19). The abdomen was the most common infection site and the admission was mainly surgical, both in S and in NS. The SOFA score did not change in both groups during the administration of eIG; however, after its termination, the SOFA continued to improve in S but worsened in NS (Table 2). The antibiotic treatment was adequate in >80% of patients of both groups. The septic shock was caused by MDR germs in 10% of S and in 21% of NS, respectively.

**Table 1:** Patients' characteristics at admission.

**Variable**	**Survivors**	**Non-survivors**	***p***
*N*	40	14	
Age, median (IQR)	69 (60.25–73)	67 (61.25–77.00)	n.s.
Male	31	23	n.s.
SAPS (mean ± SD)	41.1 ± 11.7	58.3 ± 16.4	0.0003
PCT, median (IQR) r.v. <0.5 ng/ml	48.8 (17.9–93.0)	8.4 (4.2–22.4)	0.01
CRP, median (IQR) r.v. <5 mg/L	23.0 (16.0–32.2)	20.4 (14.9–32.9)	0.07
**ADMISSION**			
Medical	5	5	0.10
Surgical	26	5	0.06
Transferred from other ICU	1	1	0.45
Emergency department	8	3	1.0
**SOURCE OF SEPSIS**			
GI tract	25	5	0.11
Urinary tract	8	5	0.28
Skin and soft tissues	2	2	0.27
Lung	1	1	0.45
Cancer	0	1	0.26
Heart	1	0	1.0
Prosthesis	1	0	1.0
Unknown	2	0	1.0

*r.v., reference values; PCT, procalcitonin; CRP, C-reactive protein*.

**Table 2:** Variations of the SOFA Score during the study period.

**Time**	**S**	**NS**	***p***
D0	12 ± 4	13 ± 5	0.7781
D1	12 ± 4	12 ± 4	0.6724
D2	11 ± 11	12 ± 13	0.26
D3	10 ± 9	11 ± 12	0.24
D4	9 ± 3	9 ± 3	0.9784
D5	7 ± 7	13 ± 13	0.0009
D6	7 ± 7	13 ± 13	0.0003
D7	6 ± 3	16 ± 9	0.0001
D8	6 ± 3	16 ± 9	0.0001
D9	6 ± 3	16 ± 9	0.0001

CLI was similar between S and NS at D0 and significantly decreased at D4 in the former group (Table 3).

**Table 3:** Time course of CLI.

**CLI, median (IQ range)**	**Survivors**	**Non-survivors**	***p***
Day 0	102 (86–144)	109 (55–212)	n.s.
Day 4	53 (21–78)	64 (22–101)	n.s.
*p* D0–D4	<0.001	0.051	

*Data are expressed as mean and IQR (25th−75th)*.

Ig values before starting eIg infusion were slightly below normal level in both groups; in particular, IgM were low in 23% of S vs. 22.5% in NS, IgA were low in 7.5% in both S and NS, and IgG were low in 46.2% of S and 57.5% of NS.

The time course of the blood values of Ig in S and NS is reported in Table 4: at D0, the Ig concentration was similar in S and NS except for IgA that were significantly higher in NS. At D4, IgG and IgM increased in both groups but only IgA were significantly higher in NS; at D7, IgM were non-significantly higher in the S (*p* = 0.05), whereas IgA were significantly higher in NS (*p* = 0.02).

**Table 4:** Variation of blood levels of Ig [data are expressed as mean and IQR (25th−75th)].

**Class**	**Day**	**Survivors**	**Non-survivors**	***p***
IgM	0	56 (41–73)	82 (43–114)	0.25
(r.v. 40–230 mg/dl)	1	94 (74–110)	105 (58–119)	0.85
	4	125 (105–150)	129 (108–138)	0.96
	7	152 (105–214)	79 (69–82)	0.05
IgA	0	153 (116–228)	245 (180–314)	0.04
(r.v. 70–400 mg/dl)	1	225 (174–331)	317 (201–349)	0.46
	4	252 (214–323)	438 (304–445)	0.02
	7	269 (223–346)	441 (347–482)	0.04
IgG	0	679 (497–874)	823 (447–955)	0.61
(r.v. 700–1,600 mg/dl)	1	1,221 (950–1,376)	1,086 (785–1,320)	0.36
	4	1,307 (1,122–1,699)	1,538 (1,248–1,774)	0.39
	7	981 (913–1,565)	1,315 (1,085–1,339)	0.4

*r.v., reference values*.

**Figure 1:**
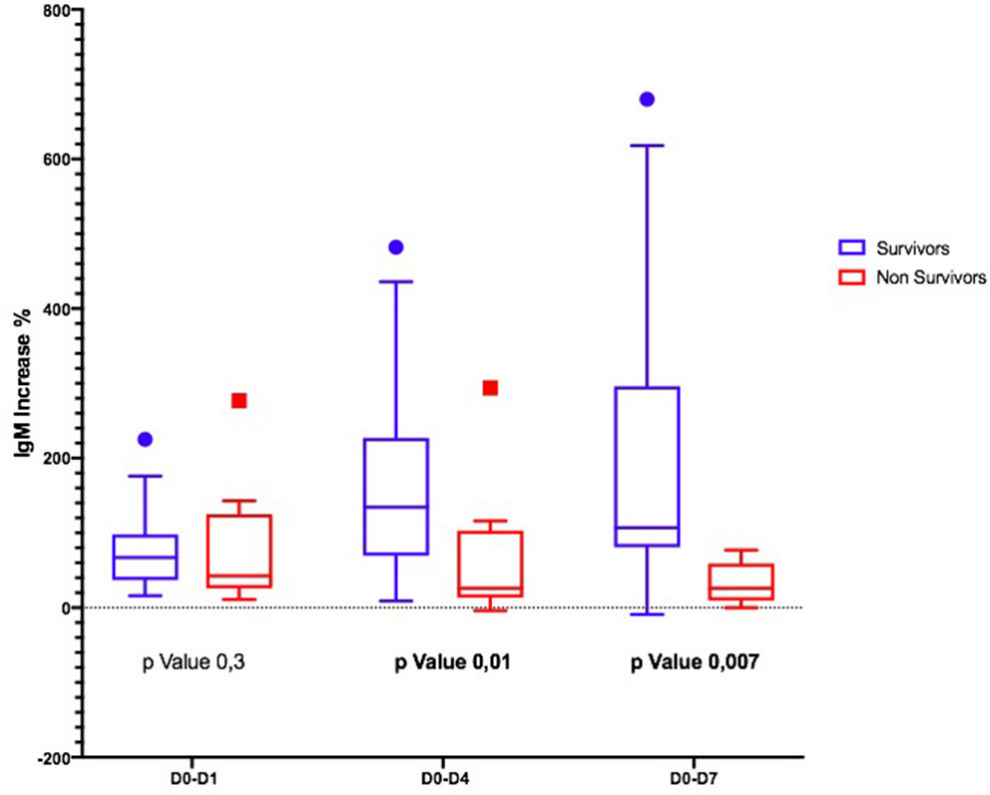
Differences in IgM variation between survivors and non-survivors.

**Figure 2:**
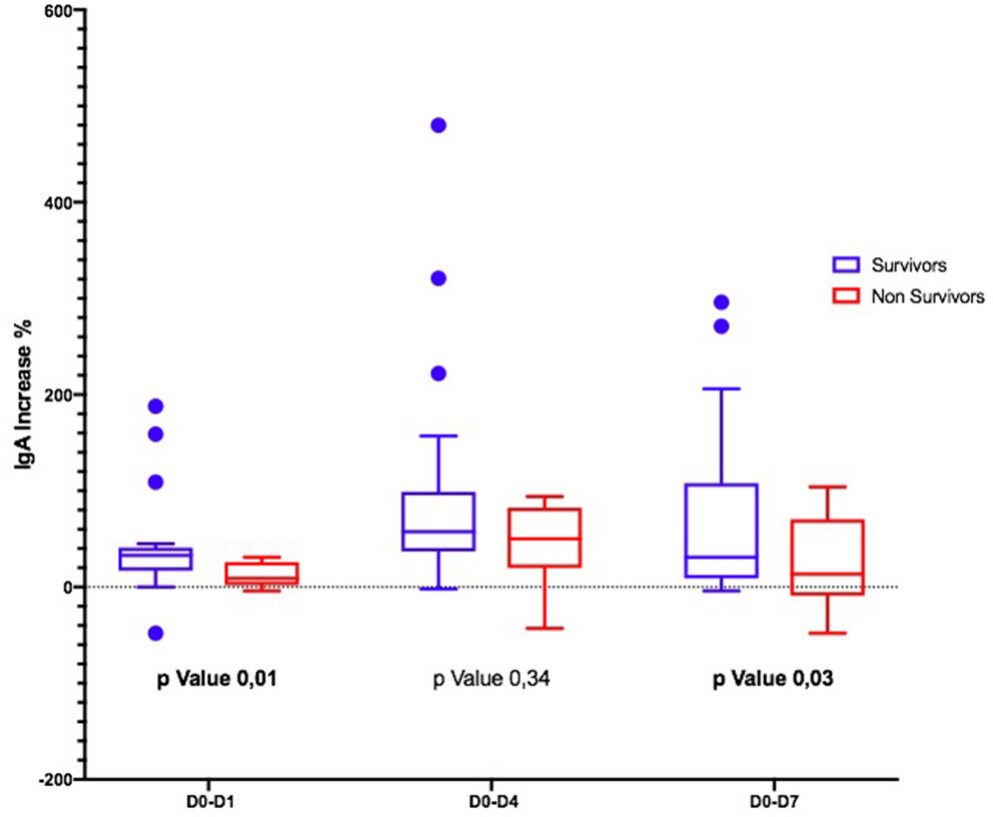
Differences in IgA variation between survivors and non-survivors.

**Figure 3:**
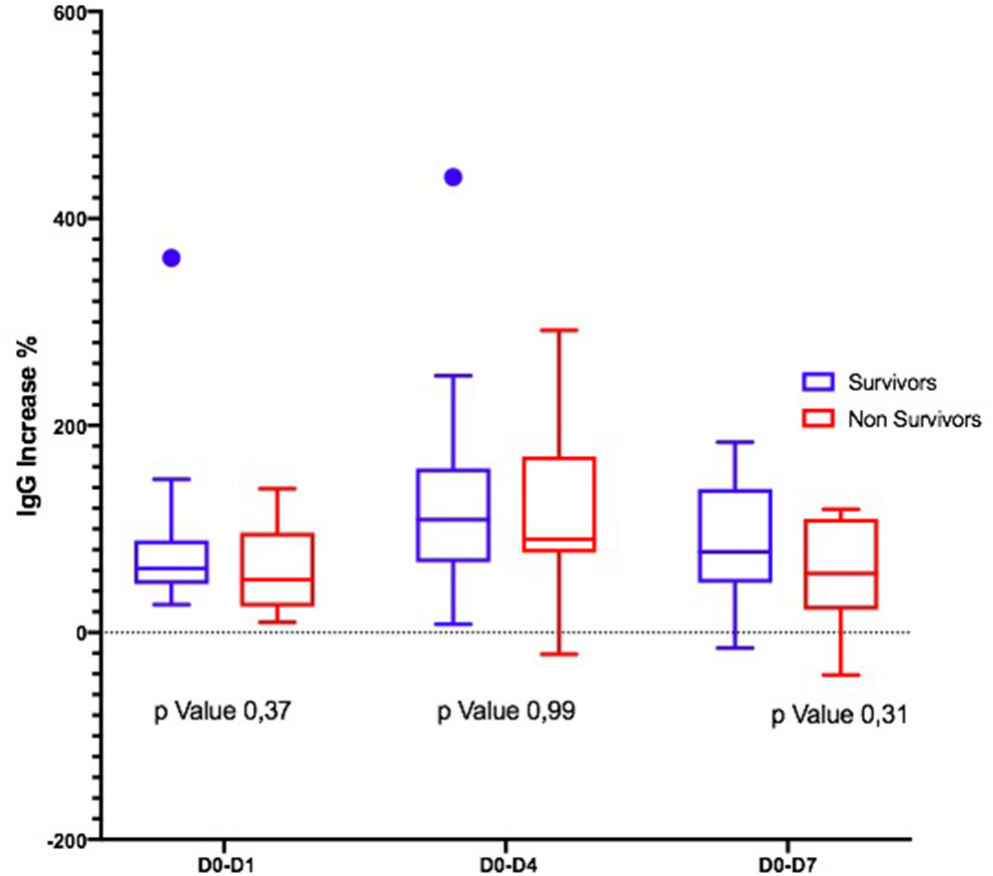
Differences in IgG variation between survivors and non-survivors.

Considering the Ig variation during the days, in D0–D4 and D0–D7, IgM and IgA increased significantly only in S (Figures 1, 2); the IgG increased but non-significantly in both groups (Figure 3).

## Discussion

A number of recent observational studies and meta-analyses demonstrated that in septic shock patients, the administration of eIg (a) is associated with a higher survival (6–8), (b) is effective also when MDR strains are involved (6, 19), but (c) is likely time-dependent, as the mortality increases by ~6% for each day of delay following the onset of septic shock even when the antibiotic treatment is appropriate (19, 20). It is conceivable that these results can be ascribed to the enhancement and/or the restoration of patients' adaptive immune capabilities and, consequently, that a threshold level of native circulating γ-globulins could exist, below which either the morbidity or mortality of septic patients could increase.

Actually, although abnormally low levels of Ig are clearly pathological, their “safe” threshold concentration in septic shock has not been identified yet. Indeed, different investigations were addressed toward the relationship between the blood levels of native Ig and the outcome of septic patients, but the results are somewhat controversial: whereas some investigators found that either isolated or combined low levels of IgG, IgM, and IgA were associated with a decreased survival (16, 21–23) and Giomarellos-Bourboulis et al. (7) showed that the progress from severe sepsis to septic shock and death was marked by decreased blood levels of IgM, other authors reported different results: in a recent meta-analysis, Shankar-Hari et al. (24) demonstrated that low levels of IgG and IgM in septic patients were not associated with a poor outcome; the same author found an inverse correlation between low IgG and elevated free light chain λ levels at the ICU admission, but these changes did not influence the outcome (25). Moreover, IgM appear to play a role also in non-septic conditions, as higher levels of this molecule have been found in survivors of a group of non-septic critically ill patients as compared with non-survivors (26).

Thus, if the relationship between endogenous Ig and the outcome of patients with septic shock is far from clear, even less is known about the kinetics of γ-globulins in patients given IvIg and their possible influence on the survival. Indeed, to clarify the relationship (if any) between the pre-treatment levels of γ-globulins, their variations during and after the IvIg administration and the outcome could help to identify the best candidate for this treatment. With this aim, this issue has been studied in two non-recent investigations: in the first, performed in septic patients randomized to receive IvIg at the dose of 900 mg/kg of body weight or placebo during 2 days, the blood values of IgG were in the normal range at admission and increased only in the treatment group but without any effect on the mortality; similar results have been reported in the second study, which involved cardiac surgery patients developing post-operative sepsis and treated with the same amount of IvIg (27, 28).

Our results obtained in patients given eIg only partly confirm previous investigation but add other pieces of information.

First, NS presented higher initial levels of γ-globulins, although this difference was significant only for the IgA. In S, the higher PCT at admission likely reflects a more pronounced natural immune response that appears blunted in NS (29, 30).

Second, the trajectory of γ-globulins during and immediately thereafter the administration of eIg was different between S and NS: whereas the IgG and the IgA increased in both groups even if less markedly in NS, in the S group at D4, the IgM more than doubled their initial values and almost tripled at D7. This finding contrasts what has been demonstrated in septic shock patients studied along a 4-week period by Giamarellos-Bourboulis et al. (7) who observed that (a) in S, the levels of native IgM sharply increased for the first 4 days but returned at baseline values after 6 days and remained stable till the 28th day; (b) conversely in NS, the IgM presented only minor fluctuations during the 4 weeks following the onset of sepsis; and (c) at the 28th day, the IgM levels of both groups overlapped. In our patients, different factors could have been responsible for the higher levels of IgM at D7 in S, including (a) an increased production of endogenous IgM, (b) the reduction of the CLI favoring the retention of these molecules into the bloodstream, and (c) their reduced consumption attributable to the decreased bacterial or PAMP load. Conversely, in NS, the ongoing production of endogenous IgG and IgA possibly associated with the reduced production and/or the consumption of IgM in NS.

Finally, during the D1–D3 interval, the differences of the circulating Ig were not accompanied by similar changes of the cumulative SOFA score despite a modest increase in NS at D7; actually, the SOFA increased in NS and remained stable in NS, confirming the findings of Vincent et al. who observed persisting rather than worsening organ failures in patients dying due to septic shock (31).

Different mechanisms can account for the different time courses of γ-globulins between S and NS, including (a) the ongoing production of endogenous IgG and IgA possibly associated with the reduced production and/or the consumption of IgM in NS; this could particularly apply to those patients in whom the septic shock was caused by MDR germs; (b) a higher pathogen and/or PAMP load and the consequent increased opsonization and clearance of the IgM molecule; and (c) the leaking from the bloodstream into the interstitial space of IgM through a more permeable capillary endothelium as suggested by their higher CLI in NS (32, 33). Actually, to overcome the relented or failed increase of the involved Ig as well as the time-dependent effect on the outcome (19) in septic shock patients, recently, Nierhaus et al. (34) suggested a different approach, basically consisting in the administration in the first 6 h of dosages higher than usual possibly followed by an infusion that could be titrated on the blood levels of the IgA and IgM. A prolonged course could be warranted particularly in patients who develop a MDR-associated septic shock after a prolonged ICU admission.

Our study has some limitations. First, due to the limited number of patients enrolled, this must be considered more a hypothesis-generating study than an investigation carrying definitive results.

Second, as it aimed only to track the variations of IgM and IgA, we did not measure other relevant immunologic variables, including the number and the classes of lymphocytes and the subclasses of IgG; these variables could and should be evaluated in a larger population of septic shock patients.

## Conclusions

We observed a different Ig trajectory between S and NS, which could be related either to (a) different capillary permeability in the two groups or to (b) higher Ig consumption in NS.

We do not have enough evidence to set a target Ig value to achieve but, looking at these results, we can try to identify a specific target population that can benefit from higher dosages of eIg, namely, those patients whose IgM and IgA values fail to increase after the first 3 days of treatment. In these subjects, a prolonged administration of eIg could be valuable.

Due to lack of high-quality evidence to support the widespread use of eIg as adjunctive therapy for sepsis, large-scale high-quality RCTs are warranted to confirm the benefits of eIg in the treatment of sepsis syndrome and to define the specific target population and the correct eIg dose.

## Data Availability Statement

The raw data supporting the conclusions of this article will be made available by the authors, without undue reservation.

## Ethics Statement

Ethical review and approval was not required for the study on human participants in accordance with the local legislation and institutional requirements. The patients/participants provided their written informed consent to participate in this study.

## Author Contributions

SZ contributed to the final statistical evaluation. All authors contributed to the data gathering, their elaboration and the preparation of the manuscript.

## Conflict of Interest

The authors declare that the research was conducted in the absence of any commercial or financial relationships that could be construed as a potential conflict of interest.

## References

[1] Busani S Damiani E Cavazzuti I Donati A Girardis M. Intravenous immunoglobulin in septic shock: review of the mechanisms of action and meta-analysis of the clinical effectiveness. Minerva Anestesiol. (2016) 82:559–72. 26474267

[2] Turgeon AFF Hutton B Fergusson DA McIntyre L Tinmouth AA Cameron DW. Meta-analysis: intravenous immunoglobulin in critically ill adult patients with sepsis. Ann Intern Med. (2007) 146:193–203. 10.7326/0003-4819-146-3-200702060-00009 17283351

[3] Cui J Wei X Lv H Li P Chen Z Liu G. The clinical efficacy of intravenous IgM-enriched immunoglobulin (pentaglobin) in sepsis or septic shock: a meta-analysis with trial sequential analysis. Ann Intensive Care. (2019) 9:27–41. 10.1186/s13613-019-0501-3 30725235 PMC6365591

[4] Rhodes A Evans LE Alhazzani W Levy MM Antonelli M Ferrer R. Surviving sepsis campaign: international guidelines for management of sepsis and septic shock: 2016. Crit Care Med. (2017) 45:486–552. 10.1097/CCM.0000000000002255 28098591

[5] Almansa R Tamayo E Andaluz-Ojeda D Nogales L Blanco J Eiros JM. The original sins of clinical trials with intravenous immunoglobulins in sepsis. Crit Care. (2015) 19:90–3. 10.1186/s13054-015-0793-0 25882822 PMC4343266

[6] Cavazzuti I Serafini G Busani S Rinaldi L Biagioni E Buoncristiano M. Early therapy with IgM-enriched polyclonal immunoglobulin in patients with septic shock. Intensive Care Med. (2014) 40:1888–96. 10.1007/s00134-014-3474-6 25217146

[7] Giamarellos-Bourboulis EJ Tziolos N Routsi C Katsenos C Tsangaris I Pneumatikos I. Improving outcomes of severe infections by multidrug-resistant pathogens with polyclonal IgM-enriched immunoglobulins. Clin Microbiol Infect. (2016) 22:499–506. 10.1016/j.cmi.2016.01.021 26850828

[8] Neilson AR Burchardi H Schneider H. Cost-effectiveness of immunoglobulin M-enriched immunoglobulin (Pentaglobin) in the treatment of severe sepsis and septic shock. J Crit Care. (2005) 20:239–49. 10.1016/j.jcrc.2005.03.003 16253792

[9] Späth PJ. Structure and function of immunoglobulins. Sepsis. (1999) 3:197–218. 10.1023/A:1009899803032

[10] Bunker JJ Erickson SA Flynn TM Henry C Koval JC Meisel M. Natural polyreactive IgA antibodies coat the intestinal microbiota. Science. (2017) 358:320–33. 10.1126/science.aan6619 28971969 PMC5790183

[11] Okai S Usui F Yokota S Hori-I Y Hasegawa M Nakamura T. High-affinity monoclonal IgA regulates gut microbiota and prevents colitis in mice. Nat Microbiol. (2016) 1:16103. 10.1038/nmicrobiol.2016.103 27562257

[12] Ehrenstein MR Notley CA. The importance of natural IgM: scavenger, protector and regulator. Nat Rev Immunol. (2010) 778–86. 10.1038/nri2849 20948548

[13] Walpen AJ Laumonier T Aebi C Mohacsi PJ Rieben R. Immunoglobulin M-enriched intravenous immunoglobulin inhibits classical pathway complement activation, but not bactericidal activity of human serum. Xenotransplantation. (2004) 11:141–8. 10.1046/j.1399-3089.2003.00098.x 14962276

[14] Tha-In T Bayry J Metselaar HJ Kaveri SV Kwekkeboom J. Modulation of the cellular immune system by intravenous immunoglobulin. Trends Immunol. (2008) 29:608–15. 10.1016/j.it.2008.08.004 18926775

[15] Venet F Gebeile R Bancel J Guignant C Poitevin-Later F Malcus C. Assessment of plasmatic immunoglobulin G and M levels in septic shock patients. Internat Immunoprharmacol. (2011) 11:2086–90. 10.1016/j.intimp.2011.08.024 21924385

[16] Giamarellos-Bourboulis EJ Apostolidou E Lada M. Kinetics of circulating immunoglobulin M in sepsis: relationship with final outcome. Crit Care. (2013) 17:R247–56. 10.1186/cc13073 24144038 PMC4056013

[17] Taccone FS Stordeur P De Backer D Creteur J Vincent JL. Gamma globulin levels in patients with community-acquired septic shock. Shock. (2009) 32:379–85. 10.1097/SHK.0b013e3181a2c0b2 19295479

[18] Singer M Deutschmann CS Seymour CW Shankar-Hari M Annane D Bauer M. The Third International Consensus Definitions for Sepsis and Septic Shock (Sepsis-3). JAMA. (2016) 315:801–10. 10.1001/jama.2016.0287 26903338 PMC4968574

[19] Berlot G Vassallo MC Busetto N Nieto Yabar M Istrati T Baronio S. Effects of the timing of administration of IgM- and IgA-enriched intravenous polyclonal immunoglobulins on the outcome of septic shock patients. Ann Intensive Care. (2018) 8:122–31. 10.1186/s13613-018-0466-7 30535962 PMC6288102

[20] Berlot G Vassallo MC Busetto N Bianchi M Zornada F Rosato I. Relationship between the timing of administration of IgM and IgA enriched immunoglobulins in patients with severe sepsis and septic shock and the outcome: a retrospective analysis. J Crit Care. (2012) 27:167–71. 10.1016/j.jcrc.2011.05.012 21737236

[21] Martin-Loeches I Muriel-Bombin A Ferrer R Artigas A Sole-Violan J Lorente L. The protective association of endogenous immunoglobulins against sepsis mortality is restricted to patients with moderate organ failure. Ann Intensive Care. (2017) 7:44–53. 10.1186/s13613-017-0268-3 28429310 PMC5399013

[22] Bermejo-Martin JF Giamarellos-Bourboulis EJ. Endogenous immunoglobulins and sepsis: new perspectives for guiding replacement therapies. Int J Antimicrob Agents. (2015) 46:S25–8. 10.1016/j.ijantimicag.2015.10.013 26597932

[23] Myrianthefs PM Boutzouka E Baltopoulos GJ. Gamma globulin levels in patients with community-acquired septic shock. Shock. (2010) 33:556–7. 10.1097/01.shk.0000370606.30525.21 20395773

[24] Shankar-Hari M Culshaw N Post B Tamayo E Andaluz-Ojeda D Bermejo-Martín JF. Endogenous IgG hypogammaglobulinemia in critically ill adults with sepsis: systematic review and meta-analysis. Intensive Care Med. (2015) 41:1393–401. 10.1007/s00134-015-3845-7 25971390

[25] Shankar-Hari M Singer M Spencer J. Can concurrent abnormalities in free light chains and immunoglobulin concentrations identify a target population for immunoglobulin trials in sepsis? Crit Care Med. (2017) 45:1829–36. 10.1097/CCM.0000000000002627 28742550

[26] Ojeda-Andaluz D Iglesias V Bobillo F Nocito M Loma AM Nieto C. Early levels of immunoglobulin M and natural killer cells predict outcome in nonseptic critically ill patients. J Crit Care. (2013) 28:1110e7–10. 10.1016/j.jcrc.2013.06.007 23953491

[27] Werdan K Pilz G Müller-Werdan U Maas Enriquez M Schmitt DV Mohr FW. Immunoglobulin G treatment of postcardiac surgery patients with score-identified severe systemic inflammatory response syndrome–the ESSICS study. Crit Care Med. (2008) 36:716–23. 10.1097/01.CCM.0B013E3181611F62F 18091548

[28] Schedel I Dreikhausen U Newtig B Höckenschnieder M Rauthmann D Balikcioglu S. Treatment of gram negative septic shock with immunoglobulin preparation: a prospective, randomized clinical trial. Crit Care Med. (1991) 19:1104–13. 10.1097/00003246-199109000-00003 1884609

[29] Dolin HH Papadimos TJ Stepkowski S Chen X Pan ZK. A novel combination of biomarkers to Herald the onset of sepsis prior to the manifestation of symptoms. Shock. (2018) 49:364–70. 10.1097/SHK.0000000000001010 29016484 PMC5811232

[30] Sim KS Oh JY Lee EJ Hur GY Lee SH Lee SY. Serum procalcitonin for differential diagnosis of acute exacerbation and bacterial pneumonia in patients with interstitial lung disease. Am J Med Sci. (2016) 351:499–505. 10.1016/j.amjms.2016.02.029 27140709

[31] Vincent JL Nelson DR Williams MD. Is worsening multiple organ failure the cause of death in patients with severe sepsis? Crit Care Med. (2011) 39:1050–5. 10.1097/CCM.0b013e31820eda29 21317650

[32] Lee WL Slutsky AS. Sepsis and endothelial permeability. N Engl J Med. (2010) 363:689–91. 10.1056/NEJMcibr1007320 20818861

[33] Cordemans C De Laet I Van Regenmortel N Schoonheydt K Dits H Huber W. Fluid management in critically ill patients: the role of extravascular lung water, abdominal hypertension, capillary leak, and fluid balance. Ann Intensive Care. (2012) 2:S1. 10.1186/2110-5820-2-S1-S1 22873410 PMC3390304

[34] Nierhaus A Berlot G Kingden-Milles D Müller E Girardis M. Best-practice IgM and IgA-enriched immunoglobulin use in patients with sepsis. Ann Intensive Care. (2020) 10:132. 10.1186/s13613-020-00740-1 33026597 PMC7538847

